# Book Notices

**Published:** 1870-03

**Authors:** 


### Book Notices.

On the Wasting Diseases of Infants and Children. By EUSTACE SMITH, M.D., London; Member of the Royal College of Physicians, etc., etc., etc. Philadelphia: Henry C. Lea. 1870.

This is a neatly published octavo volume of 195 pages. It contains an introductory chapter of 25 pages, a chapter on simple atrophy, from insufficient nourishment, one on chronic diarrhœa, one on chronic vomiting, one on rickets, on congenital syphilis, on worms, on chronic tuberculosis, on chronic pulmonary phthisis, and on tuberculization of glands. These several subjects are considered at length and with ability.

For sale at S. C. Griggs & Co.'s, Chicago. Price $1.50.

Obstetric Aphorisms, for the Use of Students commencing Midwifery Practice. By JOSEPH GRIFFITHS SWAYNE, M.D.;

Physician Accoucher to the Bristol General Hospital, and Instructor in Obstetric Medicine in the Bristol Medical School. From the Fourth Revised English Edition, with Additions by EDWARD R. HUTCHINS, M.D. Philadelphia: Henry C. Lea. 1870. Duodecimo; pp. 177. Price $1.25.

We have received the above neat and convenient little volume, through the bookstore of S. C. Griggs & Co., of this city. It is a very convenient little manuel for reference, particularly for students and young practitioners; and the fact that it has passed through four English editions, is evidence that it possesses positive merits.

